# Hydrogen sulfide reduces kidney injury due to urinary-derived sepsis by inhibiting NF-κB expression, decreasing TNF-α levels and increasing IL-10 levels

**DOI:** 10.3892/etm.2014.1781

**Published:** 2014-06-12

**Authors:** XIAN CHEN, WUJUN XU, YI WANG, HONGMEI LUO, SUQIN QUAN, JING ZHOU, NING YANG, TAO ZHANG, LEI WU, JUN LIU, XIANGYANG LONG, NENG ZHU, HUANG XIE, ZHIGANG LUO

**Affiliations:** 1 Department of Urology, Second Affiliated Hospital of University of South China, Hengyang, Hunan 421001, P.R. China; 2 Department of Histology and Embryology, University of South China, Hengyang, Hunan 421001, P.R. China

**Keywords:** hydrogen sulfide, kidney injury, urinary-derived sepsis, NF-κB, TNF-α, IL-10

## Abstract

The present study aimed to investigate the effect of hydrogen sulfide (H_2_S) on kidney injury induced by urinary-derived sepsis. Rabbits were randomly divided into control, sham, sepsis, NaHS 2.8 μmol/kg and NaHS 8.4 μmol/kg groups, with six rabbits in each group. Upper urinary tract obstruction and acute infection was induced to establish the sepsis model. Blood was collected to carry out a white blood cell (WBC) count, and creatinine (Cr) and blood urea nitrogen (BUN) analysis. Morphological changes were observed by hematoxylin and eosin (H&E) staining and transmission electron microscopy. Immunohistochemical staining was used to detect the expression levels of tumor necrosis factor (TNF)-α, interleukin (IL)-10 and nuclear factor κ-light-chain-enhancer of activated B cells (NF-κB). Cystathionine-γ-lyase (CSE) activity was measured by the spectrophotometric methylene blue method and the blood H_2_S concentration was measured by deproteinization. WBC, Cr and BUN levels were significantly elevated in the sepsis group compared with those in the control group (P<0.05). Following treatment with NaHS, the WBC, Cr and BUN levels were significantly decreased in the NaHS groups compared with those in the sepsis group (P<0.05). The pathological features of kidney injury were also alleviated by NaHS. In the sepsis group, the levels of TNF-α, IL-10 and NF-κB were significantly increased compared with those in the control group (P<0.05). In the NaHS groups, the TNF-α and NF-κB levels were significantly reduced whereas the IL-10 level was significantly increased compared with the respective levels in the sepsis group (P<0.05). The H_2_S concentration was significantly decreased in the sepsis group and this reduction was attenuated in the NaHS groups (P<0.05). Furthermore, the NaHS 8.4 μmol/kg dose revealed a more potent effect than the NaHS 2.8 μmol/kg dose. Thus, exogenous H_2_S reduced kidney injury from urinary-derived sepsis by decreasing the levels of NF-κB and TNF-α, and increasing the level of IL-10.

## Introduction

Urinary-derived sepsis is closely associated with the systemic inflammatory response syndrome (SIRS). It is caused by direct damage from bacterial toxins and the subsequent excessive body defense following bacterial infection in the urinary system. Bacterial infection in the urinary tract is most commonly caused by *Escherichia coli* (1). It leads to urinary-derived sepsis, which is a severe condition among urinary infections. The kidneys are one of the critical organs that are prone to multiple organ dysfunction syndrome (MODS) (2). Previous studies demonstrated (3,4) that during sepsis, repeated stimulation of the kidneys led to the production of a large number of pro-inflammatory cytokines [including tumor necrosis factor (TNF)-α, interleukin (IL)-6 and nuclear factor κ-light-chain-enhancer of activated B cells (NF-κB)], followed by the rapid release of large amounts of anti-inflammatory cytokines [including IL-10 and transforming growth factor β (TGF-β)]. Following this, peak concentrations of proinflammatory cytokines and anti-inflammatory cytokines were observed in the blood circulation. Early and effective regulation of the inflammatory response and the timely intervention during SIRS and sepsis is important for the prevention and treatment of MODS (5).

Hydrogen sulfide (H_2_S) may be generated endogenously. Following processing by cystathionine-β-synthase (CBS) and cystathionine-γ-lyase (CSE), sulfur-containing amino acids are able to generate H_2_S (6). It has been revealed that endogenous H_2_S is extensively involved in many physiological and pathological processes (7,8). CBS and CSE are expressed in kidney tissues and, thus, endogenous H_2_S is also produced, which plays a significant role in regulating renal functions (9). H_2_S is also important in regulating immune responses. A study by Pan *et al* revealed that NaHS (a H_2_S donor) inhibited the inflammatory response of endothelial cells to lipopolysaccharides (10). Tokuda *et al* observed that the inhalation of H_2_S was able to reduce endotoxin-induced systemic inflammation (11). Furthermore, a study by Ang *et al* demonstrated that H_2_S was able to reduce sepsis-induced acute lung injury and inflammation (12).

NF-κB is an important intracellular signaling molecule in signal transduction. NF-κB plays a significant role in sepsis and organ failure (4). It regulates a variety of genes involved in infection-related immune responses, plays an important role in inflammation and leads to organ dysfunction and mortality in patients with sepsis. Further evidence has demonstrated that H_2_S is able to inhibit the NF-κB signaling pathway (13) and regulate the expression and activity of NF-κB (14).

In the present study, an upper urinary tract infection that caused sepsis was established in rabbits by inducing acute upper urinary tract obstruction and injecting *Escherichia coli*. The renal CSE activity and endogenous H_2_S concentration in the renal tissue of the septic rabbits was observed in order to determine the association between kidney injury induced by urinary-derived sepsis and endogenous H_2_S. NaHS was used as a donor for H_2_S in order to observe the effect of exogenous H_2_S on kidney injury and NF-κB expression in renal tissue. The possible mechanism underlying the role of H_2_S in the prevention of kidney injury induced by urinary-derived sepsis was further analyzed.

## Materials and methods

### Animals and reagents

Thirty healthy male rabbits, weighing between 1.80–2.20 kg, were obtained from the Experimental Animal Center of the University of South China (Henyang, China). They were kept in standard conditions with free access to food and water. All animal experiments were conducted according to the ethical guidelines of the University of South China.

*Escherichia coli* (ATCC 25922) was provided by the Department of Microbiology of the Second Affiliated Hospital of University of South China (Henyang, China). NaHS was purchased from Sigma (St. Louis, MO, USA). TNF-α, IL-10 and NF-κB antibodies were obtained from Beijing Biosynthesis Biotechnology, Co., Ltd. (Beijing, China). Rabbit SP-HRP (streptavidin-biotin-peroxidase) kit and 3,3′-diaminobenzidine (DAB) chromogenic kit were provided by Beijing Kangwei Century Biotech Co., Ltd. (Beijing, China).

### Sepsis model establishment

Rabbits were randomly divided into five groups: control, sham, sepsis, NaHS 2.8 μmol/kg and NaHS 8.4 μmol/kg groups, with six rabbits in each group. In the control group, the rabbits were kept in standard conditions without any treatment. In the sham group, the rabbits were anesthetized through intraperitoneal injection with 10% chloral hydrate (3 ml/kg) and an incision was made through the left rectus abdominis. The middle section of the left ureter was separated and the incision was sutured. In the sepsis group, the middle section of the left ureter was separated and ligated to form an acute upper urinary tract obstruction. A suspension of *Escherichia coli* (1 × 10^8^/ml; 0.5 ml/kg) was injected into the ureter with distal ligation. The surgical procedure in the NaHS 2.8 μmol/kg and NaHS 8.4 μmol/kg groups was the same as that in the sepsis group. NaHS (50 mmol/l) was administered postoperatively through an injection into the ear vein at doses of 2.8 and 8.4 μmol/kg in the NaHS 2.8 μmol/kg and NaHS 8.4 μmol/kg groups, respectively. Rabbits were kept in standard conditions following the surgery. Left kidney tissue samples were collected at 72 h following surgery.

### Blood routine examination and renal function test

At 24 h prior to surgery and 24, 48 and 72 h following surgery, the white blood cell (WBC) count was determined by automatic cell counting. Renal function was analyzed by measuring the levels of creatinine (Cr) and blood urea nitrogen (BUN). To do this, 5 ml venous blood was collected and centrifuged at 1,629 × g for 10 min. The supernatant was analyzed using an Olympus AU800 automated biochemical analyzer (Olympus, Tokyo, Japan).

### Hematoxylin and eosin (H&E) staining

Kidney tissues were cut into 0.5-mm^3^ sections, fixed, embedded in paraffin and further cut into smaller tissue sections. The tissue sections were dewaxed in xylene and rehydrated in graded alcohols. Following washing, the sections were stained with hematoxylin and following a second wash, the sections were differentiated. The sections were subsequently stained with eosin after washing. Following dehydration and differentiation in alcohol, the sections were mounted and observed by microscopy (Eclipse E200; Nikon, Tokyo, Japan).

### Transmission electron microscopy observation

Pathological changes in the kidney tissues were observed by transmission electron microscopy. The kidney tissue was fixed in 2.5% glutaric dialdehyde for 24 h. Following rinsing three times with phosphate-buffered saline (PBS), the kidney tissue was treated with 2% osmium tetroxide for 2 h. It was subsequently dehydrated in a graded series of acetones after washing with PBS. Following dehydration, the kidney tissue was saturated in acetone/resin (1:1) at 37°C for 24 h, embedded in Epon, polymerized in an oven at 60°C for 24 h and cut into semi-thin sections (1 μm). The semi-thin sections were stained with toluidine blue for viewing with a light microscope (Nikon). They were subsequently cut into ultra-thin sections (500 Å) using an LKB III ultramicrotome (LKB Bromma, Sollentuna, Sweden). The ultra-thin sections were stained with lead nitrate and uranyl acetate, and examined with a transmission electron microscope H7500 (Hitachi, Tokyo, Japan).

### Immunohistochemical staining

Expression of TNF-α, IL-10 and NF-κB in renal tissue was detected by immunohistochemical staining. Paraffin-embedded renal tissue sections were dewaxed and hydrated. The tissue was subjected to antigen retrieval and 3% hydrogen peroxide was used to reduce endogenous peroxidase activity. Following blocking with normal serum, tissue sections were incubated with the primary antibodies anti-TNF-α, anti-IL-10 and anti-NF-κB (1:300 dilution) overnight at 4°C. Following rinsing with PBS, biotinylated goat anti-rabbit secondary antibody (Beijing Kangwei Century Biotech Co., Ltd.) was added and the sections were incubated at room temperature. The tissue sections were subsequently incubated with horseradish peroxidase (HRP)-labeled streptavidin at room temperature following rinsing with PBS. A DAB chromogenic reagent was subsequently added for color development. Finally, the sections were counterstained with hematoxylin, dehydrated, rendered transparent with xylene, mounted and observed under a light microscope.

### Measurement of serum H_2_S concentration

Experimental rabbits were anesthetized and 5–8 ml venous blood was collected. Following centrifugation, 0.1 ml serum sample was collected and combined with 0.5 ml 1% zinc acetate, 0.5 ml 20 mmol/l N,N-dimethyl-*p*-phenylenediamine sulfate and 0.4 ml 30 mmol/l FeCl_3_. Following incubation for 20 min at room temperature, 1 ml 10% trichloroacetic acid was added to precipitate the proteins. The absorbance of the supernatant following precipitation was measured at a wavelength of 670 nm. A standard curve was generated by serial dilution of NaHS. The H_2_S concentration was calculated based on the standard curve and was expressed in μmol/l.

### Measurement of CSE activity

Kidney tissues were ground to form a 10% (w/v) homogenate in potassium phosphate buffer (50 mmol/l, pH 6.8) at 4°C. The homogenate was centrifuged and the supernatant was collected in a conical flask. Subsequently, a 5-pyridoxal phosphate/potassium phosphate buffer solution (0.5%, pH 7.4, 100 mmol/l) and 0.5 mol/l L-cysteine was added to the reaction flask. A 1% zinc acetate solution (0.5 ml) and filter paper were added through the central absorbent hole. The conical flask was filled with nitrogen, sealed and incubated at 37°C in a water bath oscillator for 90 min. To terminate the reaction, 0.5 ml 50% trichloroacetic acid was added and the mixture was incubated at 37°C for 20 min. Upon termination of the reaction, the absorbance was measured at a wavelength of 670 nm. A standard curve was generated by serial dilution of NaHS. The H_2_S content was measured according to the standard curve. CSE activity was expressed as the amount of H_2_S generated per mg of kidney tissue in one minute, with a unit of nmol/min/mg.

### Statistical analysis

Data were processed using SPSS statistical software, version 18.0 (SPSS, Inc., Chicago, IL, USA). All parameters are presented as means ± standard deviations. One-way analysis of variance (ANOVA) was performed to compare the differences among the different groups. A paired t-test was carried out to analyze two samples taken at the same time point. Measured data were analyzed by a chi-square test. P<0.05 was considered to indicate a statistically significant difference.

## Results

### NaHS treatment reduces the WBC level in rabbits with sepsis

**Figure 1:**
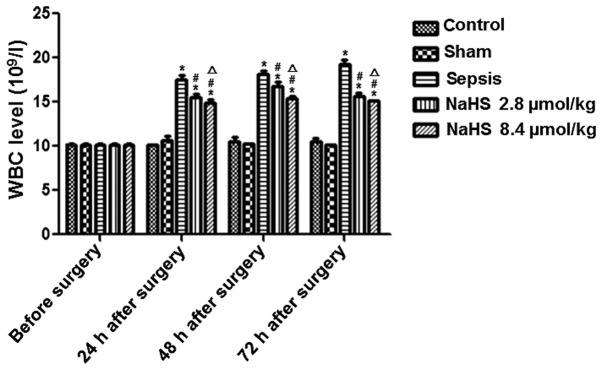
Analysis of the white blood cell (WBC) level in each group. Whole blood was collected from the control, sham, sepsis, NaHS 2.8 μmol/kg and NaHS 8.4 μmol/kg groups at 24 h prior to surgery and 24, 48 and 72 h following surgery. The WBC level was determined by automatic cell counting. Data are expressed as means ± standard deviations of three independent experiments. ^*^P<0.05, compared with the control group at the same time point; ^#^P<0.01, compared with the sepsis group at the same time point; ^Δ^P<0.05, compared with the NaHS 2.8 μmol/kg group at the same time point.

To determine the effect of NaHS on the WBC level in rabbits with urinary-derived sepsis, a sepsis model was created in the present study. The sepsis model was successfully established in the rabbits. In the sepsis group, the rectal temperature, respiratory rate and heart rate increased significantly (data not shown), and no animals died during the observation period. At 24 h prior to surgery, and 24, 48 and 72 h following surgery, the WBC level was examined and compared. The results are shown in Fig. 1. The WBC level gradually increased in the sepsis group compared with the levels in the control and sham groups, and was significantly higher at 24, 48 and 72 h following surgery (P<0.05). This result indicates that sepsis increased the WBC level in rabbits. The WBC level in the NaHS 2.8 μmol/kg and NaHS 8.4 μmol/kg groups was significantly higher than that in the control group and significantly lower than that in the sepsis group at 24, 48 and 72 h following surgery (P<0.01). Additionally, the WBC level in the NaHS 8.4 μmol/kg group was significantly lower than that in the NaHS 2.8 μmol/kg group (P<0.05). These results indicate that NaHS attenuated the sepsis-induced increase in the WBC level.

### NaHS treatment decreases Cr and BUN levels in rabbits with sepsis

**Figure 2:**
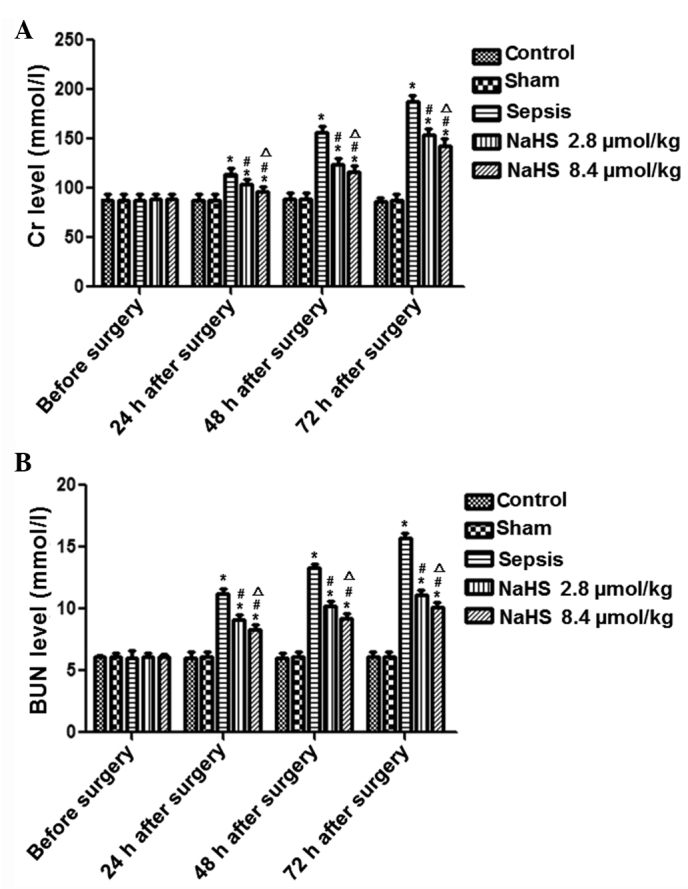
Analysis of creatinine (Cr) and blood urea nitrogen (BUN) levels in each group. To evaluate renal function, the levels of Cr and BUN were detected at 24 h prior to surgery and 24, 48 and 72 h following surgery. (A) Cr and (B) BUN levels in the control, sham, sepsis, NaHS 2.8 μmol/kg and NaHS 8.4 μmol/kg groups. Data are represented as means ± standard deviations of three independent experiments. ^*^P<0.05, compared with the control group at the same time point; ^#^P<0.01, compared with the sepsis group at the same time point; ^Δ^P<0.05, compared with the NaHS 2.8 μmol/kg group at the same time point.

To determine the effect of NaHS treatment on kidney injury induced by sepsis, the levels of Cr and BUN were examined at 24 h prior to surgery, and at 24, 48 and 72 h following surgery. The results for Cr and BUN are shown in Fig. 2. In the sepsis group, the Cr and BUN levels gradually increased with time. The Cr and BUN levels in the sepsis group were significantly higher at 24, 48 and 72 h following surgery compared with those in the control group (P<0.05). In the NaHS groups, the Cr and BUN levels gradually decreased in comparison with the levels in the sepsis group. This difference between the NaHS groups and the sepsis group was significant (P<0.01). Furthermore, there were significantly higher levels of Cr and BUN in the NaHS 8.4 μmol/kg group than in the NaHS 2.8 μmol/kg group (P<0.05). These results demonstrate that sepsis induced elevated levels of Cr and BUN in rabbits and that this elevation was inhibited by NaHS treatment.

### NaHS treatment increases the plasma H_2_S content in sepsis rabbits

**Figure 3:**
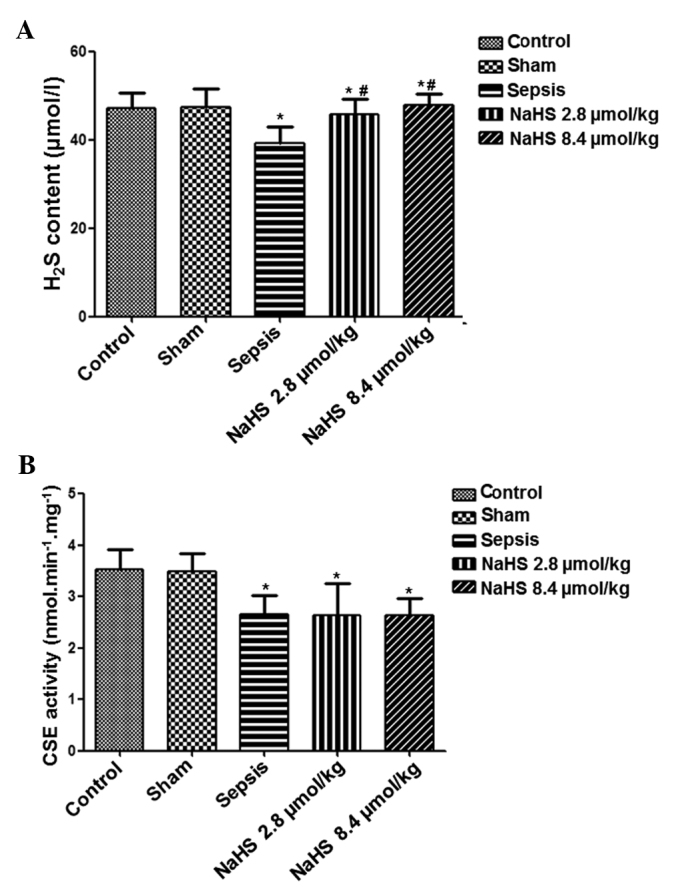
Analysis of H_2_S level in plasma and cystathionine-γ-lyase (CSE) activity in renal tissue. (A) Venous blood was collected from the control, sham, sepsis, NaHS 2.8 μmol/kg and NaHS 8.4 μmol/kg groups at 72 h following surgery and the serum H_2_S concentration was measured. (B) Renal tissue was taken at 72 h following surgery from the control, sham, sepsis, NaHS 2.8 μmol/kg and NaHS 8.4 μmol/kg groups. CSE activity was detected. Data are expressed as means ± standard deviations of three independent experiments. ^*^P<0.05, compared with the control group at the same time point; ^#^P<0.01, compared with the sepsis group at the same time point.

To analyze the effect of NaHS on endogenous H_2_S, serum samples were collected at 72 h following surgery and the plasma H_2_S content was determined. As shown in Fig. 3, plasma H_2_S content was significantly reduced in the sepsis group compared with that in the control group (P<0.05). Following treatment with NaHS, the plasma H_2_S content increased in the NaHS 2.8 μmol/kg and NaHS 8.4 μmol/kg groups. The plasma H_2_S contents in the NaHS 2.8 μmol/kg and NaHS 8.4 μmol/kg groups were significantly higher compared with that in the sepsis group (P<0.05). These data indicate that NaHS treatment increased the endogenous H_2_S levels of septic rabbits.

The CSE activity in the renal tissue was analyzed. Left renal tissue was collected at 72 h following surgery and the CSE activity was detected in each group. As shown in Fig. 3, the CSE activity in the sepsis, NaHS 2.8 μmol/kg and NaHS 8.4 μmol/kg groups was significantly lower than that in the control group (P<0.05). However, no significant difference was identified between the sepsis and NaHS 2.8 μmol/kg, sepsis and NaHS 8.4 μmol/kg, nor the NaHS 2.8 μmol/kg and NaHS 8.4 μmol/kg groups. These results suggest that in urinary-derived sepsis, the endogenous H_2_S content and CSE activity are reduced, and that NaHS treatment may increase H_2_S content but not CSE activity.

### NaHS treatment alleviates the pathological features of renal tissue in septic rabbits

**Figure 4:**
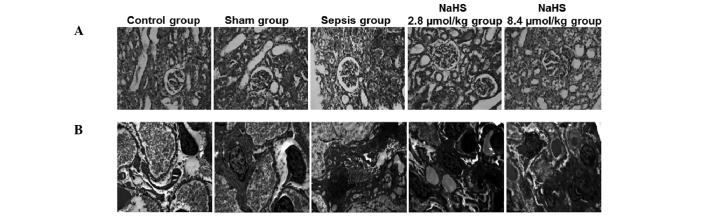
Pathological features of the renal tissue in each group. Renal tissue was collected at 72 h following surgery. The pathological features of the renal tissue were observed by hematoxylin and eosin (H&E) staining and transmission electron microscopy. Representative (A) H&E staining and (B) transmission electron microscopy results in the control, sham, sepsis, NaHS 2.8 μmol/kg and NaHS 8.4 μmol/kg groups. Magnification, ×400 and ×10,000, respectively.

To investigate the effect of NaHS on renal injury induced by sepsis, the pathological features of renal tissue were observed by H&E staining and transmission electron microscopy at 72 h following surgery. The representative H&E staining and transmission electron microscopy results are shown in Fig. 4. The renal tissue revealed normal morphology in the control and sham groups. There was no congestion, edema or inflammatory cell infiltration. In the sepsis group, mucosal necrosis and extensive neutrophil infiltration was present in the pelvis and calyces as well as glomerular deformation and renal capsule expansion. Swelling and necrosis was observed in the renal tubular epithelial cells. The renal tubules were also enlarged and infiltrated with neutrophils. In the kidney interstitium, congestion, edema, inflammatory cell infiltration and abscess formation was observed. Following treatment with NaHS, these pathological changes were reduced in the NaHS 2.8 μmol/kg and NaHS 8.4 μmol/kg groups.

The pathological changes in renal tissue were further observed by transmission electron microscopy. As shown in Fig. 4, no abnormal structures were visible in the control and sham groups. There was no edema or vacuolation in the epithelial cells of the renal tubules and the mitochondria and endoplasmic reticulum were regularly arranged. Podocytes and mesangial cells revealed a normal morphology and the basement membrane also remained intact. In the sepsis group, edema was present in the kidney interstitium, there was extensive atrophy in the tubular epithelium and interstitial fibroblast proliferation. Mitochondria in the renal tubular epithelial cells were swollen and disorganized; their number was also reduced. Focal fusion of glomerular podocytes was observed. Furthermore, there was proliferation of the mesangial and endothelial cells. In the NaHS 2.8 μmol/kg and NaHS 8.4 μmol/kg groups, however, these pathological changes were alleviated.

Collectively, these results suggest that the pathological features of renal tissue induced by sepsis were decreased by NaHS treatment.

### NaHS increases the expression level of IL-10 and decreases the expression levels of TNF-α and NF-κB in the renal tissue of septic rabbits

**Figure 5:**
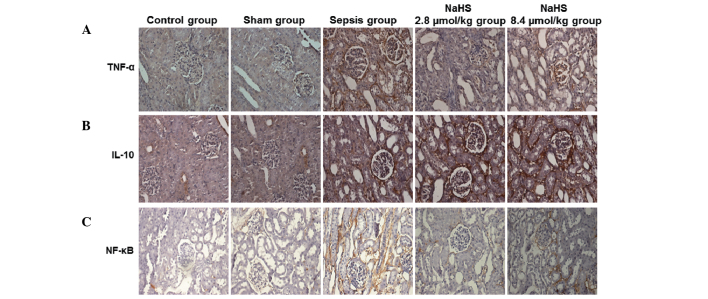
Analysis of the expression levels of tumor necrosis factor α (TNF-α), interleukin 10 (IL-10) and nuclear factor κ-light-chain-enhancer of activated B cells (NF-κB) in each group. Renal tissue was collected at 72 h following surgery. Expression levels of TNF-α, IL-10 and NF-κB were analyzed by immunohistochemistry. Representative immunohistochemical staining results for (A) TNF-α, (B) IL-10 and (C) NF-κB in the control, sham, sepsis, NaHS 2.8 μmol/kg and NaHS 8.4 μmol/kg groups. Magnification, ×400.

To analyze the possible mechanism by which H_2_S prevents kidney injury induced by urinary-derived sepsis, TNF-α, IL-10 and NF-κB protein expression in left kidney tissue was determined by immunohistochemistry at 72 h following surgery. The representative immunohistochemical results are shown in Fig. 5 and the quantitative results are shown in Table I. Cells with yellow or brown particles were positively stained cells. As shown in Fig. 5 and Table I, TNF-α, IL-10 and NF-κB proteins were weakly expressed in each group. The expression levels of TNF-α, IL-10 and NF-κB increased in the sepsis group compared with those in the control group. In the NaHS 2.8 μmol/kg and NaHS 8.4 μmol/kg groups, the expression levels of TNF-α and NF-κB were lower than those in the sepsis group. However, the expression levels of IL-10 were higher in the NaHS groups than in the sepsis group. Statistically, compared with those in the control group, the expression levels of TNF-α, IL-10 and NF-κB were significantly higher in the sepsis group (P<0.05). The expression levels of TNF-α and NF-κB were significantly lower in the NaHS groups compared with those in the sepsis group (P<0.05). Furthermore, the NaHS 8.4 μmol/kg group had significantly higher levels of TNF-α and NF-κB than the NaHS 2.8 μmol/kg group (P<0.05). By contrast, the NaHS groups had a significantly higher level of IL-10 (P<0.05). The IL-10 level in the NaHS 8.4 μmol/kg group was significantly higher than in the NaHS 2.8 μmol/kg group. These data indicate that NaHS regulated the expression of TNF-α, IL-10 and NF-κB in septic rabbits.

## Discussion

A previous study reported that ulinastatin effectively prevented urinary-derived sepsis and reduced inflammation by decreasing the level of TNF-α and increasing IL-10 (15). In patients with early sepsis-induced acute kidney injury, renal replacement therapy is effective in restoring organ function and increasing survival rate through the removal of inflammatory mediators IL-10 and IL-6 (16). In a study by Souza *et al* (4), sepsis was induced in rats and it was identified that erythropoietin prevented the acute kidney injury induced by sepsis through the inhibition of NF-κB and the upregulation of endothelial nitric oxide synthase.

Studies have demonstrated that H_2_S plays an important regulatory role in sepsis-related inflammation by inhibiting the NF-κB signaling pathway and the expression of inflammatory cytokines. A study by Li *et al* reported that by downregulating NF-κB expression, H_2_S was able to promote neutrophil aggregation to the site of inflammation, increase neutrophil migration and adhesion, reduce plasma levels of TNF, IL-1 and IL-6, and increase the level of IL-10 (17). H_2_S may also reduce inflammation by inhibiting the NF-κB/cyclooxygenase-2 (COX-2) pathways and decreasing the production of IL-1β, IL-6 and IL-8 (18). A study by Pan *et al* (19) revealed that H_2_S was able to inhibit NF-κB activation, increase the level of heme oxygenase 1 (HO-1) and reduce TNF-α-induced inflammation.

The present study revealed that endogenous H_2_S levels decreased in the renal tissue of rabbits with urinary-derived sepsis. Throughout treatment with NaHS, the levels of NF-κB and TNF-α decreased, the level of IL-10 increased, and kidney function improved. These results suggest that NaHS protected the kidneys during urinary-derived sepsis by inhibiting NF-κB, reducing the level of TNF-α and increasing the level of IL-10, thereby reducing inflammation in urinary-derived sepsis. The protective effect of 8.4 μmol/kg NaHS was more significant than that of 2.8 μmol/kg NaHS, indicating that NaHS protected the kidneys from injury in a dose-dependent manner.

## Figures and Tables

**Table I:** IL-10, TNF-α and NF-κB expression comparison in renal tissue at 72 h following surgery (OD, means ± standard deviations, n=6).

Groups	TNF-α	IL-10	NF-κB
Control	0.141±0.023	0.114±0.014	0.110±0.016
Sham	0.139±0.013	0.114±0.014	0.111±0.017
Sepsis	0.264±0.017^a^	0.225±0.015^a^	0.277±0.017^a^
NaHS 2.8 μmol/kg	0.222±0.015^a^,^b^	0.267±0.015^a^,^b^	0.265±0.017^a^,^b^
NaHS 8.4 μmol/kg	0.198±0.009^a^–^c^	0.275±0.016^a^–^c^	0.221±0.017^a^–^c^

a P<0.05, compared with the control group;

b P<0.01, compared with the sepsis group;

c P<0.05, compared with the NaHS 2.8 μmol/kg group;

TNF-α, tumor necrosis factor α; IL-10, interleukin 10; NF-κB, nuclear factor κ-light-chain-enhancer of activated B cells, OD, optical density.
